# Digit span and Bisyllabic non‐word span: Italian norms

**DOI:** 10.1111/jnp.12420

**Published:** 2025-02-26

**Authors:** Konstantinos Priftis, Daphne Gasparre, Denyse Carazzolo, Valeria Vaccaro, Roberta Toffano, Marco Pitteri, Massimo Grassi

**Affiliations:** ^1^ Department of General Psychology University of Padua Padua Italy; ^2^ Department of Translational Biomedicine and Neuroscience University of Bari Aldo Moro Bari Italy; ^3^ Department of Neuropsychology National Hospital for Neurology and Neurosurgery London UK; ^4^ Department of Neuroinflammation Queen Square Institute of Neurology, University College London London UK

**Keywords:** attention, digit span, executive functions, phonological loop, short‐term memory, working memory

## Abstract

We standardized a new version of the Digit span test and the first version of the Bisyllabic non‐word span test, both measuring the phonological loop, in an Italian sample of neurologically healthy adults (*n* = 225). All stimuli were administered to the participants through a computerized procedure to avoid the influence of the examiner on participants' performance. We used a preliminary test to exclude the presence of sensory‐perceptual and articulatory‐motor difficulties that might have influenced the results. The results revealed that both Age and Education were significant predictors of participants' performance on the Digit span test. By contrast, only Age predicted significantly participants' performance on the Bisyllabic non‐word span test. The average Digit span was approximately twice as large as the average Bisyllabic non‐word span, suggesting that the latter might be a strategy‐free measure of the phonological loop. The Bisyllabic non‐word span is an innovative and specific measure for identifying phonological short‐term memory deficits. For all tests, adjusted and equivalent scores are provided to facilitate results interpretation and clinical applicability.

## INTRODUCTION

In the current understanding of the human mind, working memory is hypothesized to be the cognitive subsystem that underpins short‐term retention and manipulation of information (Baddeley, 1986, 2003, 2012). According to Baddeley's model (Baddeley, 1986, 2003, 2012), working memory is composed by the phonological loop (i.e., phonological short‐term memory), the visuospatial sketchpad (i.e., visuo‐spatial short‐term memory), the episodic buffer, and the central executive (Baddeley, 2003). In the model, the phonological loop is the component of working memory aimed at short‐term retention of phonological information (e.g., phonemes, syllables, words, and non‐words; Baddeley, 2012). Our ability to briefly retain and manipulate phonemic sequences is important for speech processing and for the learning of the maternal language by children (Baddeley et al., 1998; Gathercole & Baddeley, 1993). Both in children and adults, the phonological loop is hypothesized to be involved in learning new terms (e.g., acronyms, neologisms, etc.), in processing syntactically complex spoken sentences (Papagno et al., 2007), and in learning foreign languages (Baddeley et al., 1998; Papagno et al., 1991).

The phonological loop is modelled as including a phonological short‐term memory buffer (i.e., the phonological store) and an articulatory rehearsal process. The latter refreshes the contents of the phonological store and converts graphemes into phonemes (Baddeley, 1986, 2003, 2012). Finally, in the model of Baddeley (1986, 2003, 2012), the central executive plays a key role when phonological information must not only be retained (phonological loop), but should also be manipulated. For instance, the repetition of a sequence of spoken number words (digits), in the same order (forward modality: 4 ‐ 1 ‐ 0 ‐ 7 → 4 ‐ 1 ‐ 0 ‐ 7), requires only retention of those digits in the phonological loop. By contrast, the repetition of the same digits, in reverse order (backward modality: 4 ‐ 1 ‐ 0 ‐ 7 → 7 ‐ 0 ‐ 1 ‐ 4), requires not only retention (phonological loop), but also the active manipulation of the digits (central executive; for a review, see Lezak, 1995).

The phonological loop finds a counterpart in the human brain through a specific cognitive‐neuroanatomical network (Fiez, 2016). The phonological store is implemented in the left inferior parietal lobule (Brodmann area 40: supramarginal gyrus; Baddeley, 2003). The articulatory rehearsal process is associated with activity in Broca's area (Brodmann areas 44–45: posterior part of the left inferior frontal gyrus; Baddeley, 2003). The phonological loop is doubly dissociated from all other components of working memory (for reviews, see Baddeley, 2003; Vallar, 2002; Vallar & Shallice, 1990). Finally, the phonological loop is doubly dissociated from all components of long‐term memory. For instance, severely amnesic patients, because of long‐term memory impairment, can show intact short‐term retention of phonological information (Baddeley, 2009). By contrast, patients with impaired short‐term retention of phonological information are not amnesic (Shallice & Warrington, 1970).

There are different neuropsychological tests to assess the phonological loop. In Italy, the most diffused tests for measuring the phonological loop are the Serial repetition of bisyllabic words test (Spinnler & Tognoni, 1987) and the Digit span test (Monaco et al., 2013; Orsini et al., 1987; Pasotti et al., 2022). On the Serial repetition of bisyllabic words test (Spinnler & Tognoni, 1987), the examinee is asked to orally repeat sequences of bisyllabic spoken words. The performance of the examinees on the Serial repetition of bisyllabic words test was positively correlated with education, and negatively correlated with age. Finally, no effects of biological sex (males vs. females) were reported. Unfortunately, Spinnler and Tognoni (1987) included only the forward modality in their standardization.

On the Digit span test, participants are required to listen to a sequence of spoken number words and, then, to repeat them orally. Different standardizations of the Digit span test are available in Italy. For instance, Orsini et al. (1987) found that participants' performance on this test was positively correlated with education, and negatively correlated with age. Finally, no effects of biological sex (males vs. females) were reported. Unfortunately, Orsini et al. (1987) included only the forward modality in their standardization.

Monaco et al. (2013) standardized a version of the Digit span test. In that standardization, both the forward and the backward modalities were included. The performance of Monaco's et al.'s sample was positively correlated with education, and negatively correlated with age. In contrast, no effects of biological sex (males vs. females) were reported. Finally, also in the latest available standardization of the Digit span (Pasotti et al., 2022), participants' performance was positively correlated with education, but negatively correlated with age. In contrast with the previous standardizations (Monaco et al., 2013; Orsini et al., 1987), biological sex was a significant predictor of performance, but only for the Digit span backward (Pasotti et al., 2022).

Some issues could be raised regarding the tests used so far to examine the phonological loop. These tests include materials (i.e., digits and words) that allow the use of strategies by the examinee, and these strategies can impair the validity of the measure. For instance, the presentation of digits could activate the participant's semantic memory, allowing for effects such as numerical grouping (units, teens, tens, hundreds, etc.). Digits can be also interconnected and chunked to create numbers that are significant for the person (historical and personal dates, public numbers, etc.).

The presentation of words can elicit the activation of semantic information and mental images, including mental visualization strategies, whereby a cognitive task that is supposed to be auditory‐verbal can actually involve multimodal features (e.g., visual). This, in turn, can facilitate the subsequent retrieval of the presented words. In any case, digits and words might not enable a veridical measure of the phonological loop, because these items can also activate long‐term memory. A possible solution is the use of non‐words (i.e., meaningless sequences of phonemes). Given that non‐words are meaningless, the examinees are prevented from activating their long‐term memory. The fact that non‐words are a less strategy‐prone measure of the phonological loop is suggested from studies in which a lower span for non‐words than for words has been reported (for a review, see Baddeley, 2003).

Another limitation of the previous tests is the presentation method, which occurs with the examiner reading aloud the items (words or digits). This type of presentation might not guarantee an identical administration for all the examinees tested by different examiners. Indeed, reading aloud depends on variables linked to the examiner's characteristics (e.g., voice's sex, voice's timber, emotional intonation, prosody, speed, voice's loudness, etc.) and to the presentation of the stimuli (e.g., duration of the stimuli, duration of the pauses between the stimuli, etc.). Thus, there is no guarantee that the characteristics of the examiner and the presentation, during the standardization process, will be the same as those of the different examiners who are going to use the test. All these factors might significantly limit the external validity regarding the standardization of the various tests measuring the phonological loop.

To avoid the aforementioned problems, computerized versions of neuropsychological tests could be the best solution. In this case, all examiner's characteristics are kept constant. A good example of using a computerized input, when presenting auditory stimuli, is the Paced Auditory Serial Addition Test (Wingenfeld et al., 1999). Furthermore, the importance of computerized assessment for controlling precision, in stimuli presentation, has been recently re‐highlighted (Harris et al., 2024). Finally, specific guidelines on how to device computerized neuropsychological tests are available (Bauer et al., 2012).

Another salient point is the absence, in the previous standardizations, of preliminary tests to guarantee the integrity of the examinees' input (sensory‐perceptual) and output (articulatory‐motor) processes. These input/output processes are necessary for interfacing with and measuring the phonological loop. For instance, in clinical practice, a patient can be unable to correctly perceive and articulate oral items. Consequently, the patient might have an apparently reduced span, not because of damage to the phonological loop, but because of sensory‐perceptual or articulatory‐motor deficits. Recent findings have suggested that sensory and motor changes may occur years before the cognitive symptoms of dementia, potentially increasing the risk of cognitive decline (Jafari et al., 2019). Therefore, conducting thorough auditory screening and early diagnosis of auditory conditions, such as age‐related hearing loss, tinnitus, or other perceptual disorders, can enable a more reliable neuropsychological assessment (Gasparre et al., 2023).

Usually, neuropsychological assessment is preceded by a clinical interview, which includes medical and preliminary cognitive screening tests. These first steps can already inform on sensory‐perceptual deficits, such as hearing impairments, asking for additional elements that the examiner should take into account (e.g., speak louder and/or provide concise instructions and questions). Nevertheless, a deterministic effect of such weaknesses on observed test performance is not straightforward and might warrant additional instrumental investigation, with special reference to rather difficult acoustic input, such as non‐words. Thus, a preliminary ad hoc test is warranted to guarantee normal acoustic processing. This preliminary test is of paramount importance when no screening testing is available (e.g., a particular research setting). This can be also the case of a very specific diagnostic question that does not take place during a comprehensive neuropsychological evaluation (e.g., “Is there a deficit with phonological short‐term memory?”). For instance, Quaranta et al. (2016) introduced a preliminary auditory discrimination task (e.g., voice discrimination), necessary for their participants to perform, thereafter, recognition tests for famous voices.

To tackle the issues raised above, we proposed a new ‐the first complete‐ standardized Italian version of the Digit span test and of the Bisyllabic non‐word span test. There were three innovative aspects in the present standardization: (a) the use of non‐words, (b) the computerized presentation, and (c) the presence of a sensory‐perceptual and articulatory‐motor preliminary test. The first novelty regards the problem of the influence of long‐term (semantic) memory on performance. To tackle this problem we proposed the use of bisyllabic non‐words. In this way, we ensured that the performance of the examinees was not (or was highly unlikely) influenced by long‐term memory.

The second novelty consisted in the implementation of a computerized administration of the tests. All the stimuli were audio‐recorded and presented to the participants through headphones. The comp+uterized administration of the tests ensured that each examinee heard the same stimuli, spoken by the same examiner having the same intonational and prosodic characteristics. The stimuli were presented with fixed speed and duration. Finally, all stimuli were presented at a fixed pace to guarantee the same duration of the pauses among them.

The third novelty of our study was the implementation of a preliminary test, useful for testing the integrity of the examinees' sensory‐perceptual (i.e., acoustic input) and articulatory‐motor processes (i.e., spoken output). Before listening to the audio‐sequences included in the tests, participants were asked to repeat aloud, one by one, all the single items that composed the audio‐sequences. To be included in the final sample, all examinees should have had an intact performance on the preliminary test. Finally, for all tests, we used the same demographic and biological predictors of performance as those employed in the previous standardizations (i.e., Education, Age, and Biological sex) to obtain adjusted scores (Monaco et al., 2013; Orsini et al., 1987; Pasotti et al., 2022; Spinnler & Tognoni, 1987).

## METHOD

### Participants

The present study was conducted according to the principles of the Declaration of Helsinki. All participants gave their written informed consent before participating in the study. The sample size for multiple linear regression with three predictors (Age, Education, and Biological sex) was calculated a priori, through GPower (Faul et al., 2007), with effect size = 0.015 (f2), alpha = .001, and power = .95. The overall required sample for reaching significance was 206 participants. We opted for a slightly larger sample of 225 participants to better control for potential dropouts and outliers. We recruited participants, according to the following inclusion criteria:

1. Adjusted score ≥24 on the Mini Mental State Examination (MMSE; Folstein et al., 1975; Magni et al., 1996; Measso et al., 1993);
2. Negative neurologic history for the presence of brain lesions or other neurological disorders (e.g., stroke, epilepsy, traumatic brain injury, dementia, tumours, etc.);
3. Negative psychiatric history (e.g., anxiety, depression, drug abuse, alcohol abuse, etc.);
4. Normal ability to comprehend task instructions (e.g., on the preliminary trials, participants responded only after all the stimuli were presented, including the second tone; see “Stimuli” section);
5. Normal ability to correctly perceive and repeat all the preliminary audio‐sequences and the examples in the experimental audio‐sequences (see “Stimuli” section).

Data collection took part in Veneto, Puglia, and Sicily (Italy). Fifty‐seven participants did not fulfil at least one of the inclusion criteria and they were excluded from the study. Two‐hundred twenty‐five healthy participants (right‐handed = 214; left‐handed = 8; ambidextrous = 3; females = 133; males = 92) fulfilled all the inclusion criteria and took part to the study (Age: *M* = 51.56 years, SD = 19.664; Range: 20–92; Education: *M* = 12.569 years; SD = 5.076; Range: 3–25). The distribution of participants as a function of Age, Education, and Biological sex is reported in Table 1.

**TABLE 1:** Distribution of the participants as a function of Age, Education, and Biological sex.

*N* (females/males)	Age intervals (years)															
	20–24	25–29	30–34	35–39	40–44	45–49	50–54	55–59	60–64	65–69	70–74	75–79	80–84	85–89	90+	Total
Education intervals (years)																
0–5	0	0	0	0	0	0	0	0	3 (1/2)	3 (1/2)	5 (3/2)	5 (3/2)	12 (8/4)	5 (4/1)	3 (0/3)	36 (20/16)
6–8	0	0	0	0	3 (2/1)	5 (2/3)	3 (1/2)	7 (3/4)	5 (4/1)	3 (1/2)	2 (1/1)	4 (2/2)	1 (0/1)	2 (1/1)	0	35 (17/18)
9–13	4 (1/3)	6 (3/3)	4 (1/3)	2 (1/1)	5 (2/3)	8 (5/3)	6 (5/1)	9 (6/3)	9 (3/6)	3 (3/0)	3 (2/1)	4 (4/0)	0	0	0	63 (36/27)
14–18	15 (12/3)	16 (10/6)	7 (3/4)	8 (5/3)	3 (1/2)	3 (2/1)	6 (3/3)	7 (4/3)	2 (2/0)	4 (2/2)	1 (1/0)	0	0	0	0	72 (45/27)
19+	0	3 (3/0)	6 (5/1)	2 (1/1)	2 (2/0)	3 (1/2)	1 (1/0)	2 (1/1)	0	1 (1/0)	0	1 (1/0)	0	0	0	19 (16/3)
Total	19 (13/6)	25 (16/9)	17 (9/8)	11 (7/4)	13 (7/6)	19 (10/9)	16 (10/6)	24 (14/10)	19 (10/9)	14 (8/6)	11 (7/4)	14 (9/5)	13 (8/5)	7 (5/2)	3 (0/3)	225

### Materials, apparatus, and stimuli

#### Materials

We designed and used a semi‐structured interview to obtain demographic data (Age, Education, Handedness, and Biological sex) and to verify the absence of any medical issues (e.g., neurologic and psychiatric disorders) related to the inclusion criteria (see “Participants” section). The semistructured interview is available in Supplemental Data 1 (https://osf.io/mna92/). To exclude participants affected by cognitive disorders, we employed the MMSE (Folstein et al., 1975). We used the standardization of Measso et al. (1993), for participants aged between 20 and 79 years, and the standardization of Magni et al. (1996) for participants aged over 79 years.

#### Apparatus

For each span test, all acoustic stimuli were delivered to the participants by means of a laptop ACER ASPIRE 7750G (Windows 7 Home Premium, RAM: 8 GB, CPU: Intel CORE i5‐2450M, p2,5 GHz Quad CORE; audio card: Realtek). All acoustic stimuli were delivered to the participants through headphones (Sennheiser), by means of lists created in *Windows Media Player*. The examiner used earphones to monitor that stimuli were delivered correctly to the participants. The intensity of the stimuli was set at a comfortable listening level and remained fixed for all the participants. More precisely, the sound volume in *Windows Media Player* was set to 100%, while the laptop's volume was set to 45%. This configuration resulted in a sound level of 72 dB SPL, which was comfortable for the listener (i.e., clearly audible, neither too loud nor too quiet).

#### Stimuli

Stimuli consisted in audio‐sequences of spoken number words (Digit span test: preliminary trials, forward modality, and backward modality) or spoken non‐words (Bisyllabic non‐word span test: preliminary trials, forward modality, and backward modality). The sheet for registering the responses of the participants is available in Supplemental Data 2 (https://osf.io/mna92/). All instructions are also available (https://osf.io/mna92/). All the audio‐sequences are available upon request to the corresponding author.

##### The Digit span test

The audio‐sequences composing the Digit span test consisted of number words spoken by a male voice (range: 1–9) and were recorded at 48 kHz sampling frequency and 24 bit resolution in a soundproof booth. Each audio‐sequence was preceded and followed by a 1 kHz pure tone (duration = 500 ms; sampling frequency 48 kHz; resolution: 24 bit). The first tone was presented 2 s before the accent of the first number word. The second tone was presented 2 s after the accent of the last number word. Spoken number words were linked together, so that there was a silent interval of 1.5 s between the accent of one number word and that of the next one.

First, nine preliminary audio‐sequences were created, consisting of a tone, a single spoken number word (either 1, 2, 3, 4, 5, 6, 7, 8, or 9), and a tone (Table 2). Subsequently, the experimental audio‐sequences were created. The length of the stimuli, within each audio‐sequence, ranged from two to nine spoken number words. Each experimental audio‐sequence consisted of a tone, the spoken number words (length range: two to nine number‐words), and a tone. The order of the spoken number words (preliminary and experimental) was pseudorandom. More in detail, we excluded repeated numbers (e.g., 2 ‐ 2 ‐ 2), sequences of multiples (e.g., 2 ‐ 4 ‐ 6), numbers in ordered magnitude (increasing; e.g., 1 ‐ 2 ‐ 3 or decreasing; e.g., 3 ‐ 2 ‐ 1), or numbers that could be easily grouped to facilitate their memorization (e.g., a sequence composed of several prime numbers).

**TABLE 2:** Spoken number words included in the Digit span test (forward and backward).

Preliminary audio‐sequences								
7	1	9	2	4	6	5	8	3

Length	Experimental audio‐sequences	
	Digit span test: Forward	Digit span test: Backward
2	2‐5 (example) 1‐7 4‐6	8‐3 (example) 9‐5 8‐6
3	2‐1‐5 3‐8‐4	1‐7‐9 1‐9‐3
4	6‐8‐3‐9 2‐1‐7‐9	1‐4‐6‐3 7‐3‐5‐2
5	3‐4‐9‐6‐8 7‐9‐2‐8‐1	7‐2‐6‐3‐8 2‐5‐1‐4‐6
6	9‐2‐5‐8‐7‐3 1‐7‐5‐2‐6‐8	5‐4‐8‐6‐2‐9 3‐9‐5‐1‐6‐2
7	7‐9‐6‐4‐2‐5‐8 6‐4‐7‐2‐8‐5‐3	2‐1‐4‐8‐3‐7‐5 1‐5‐8‐4‐6‐9‐7
8	9‐7‐3‐2‐8‐4‐5‐1 1‐4‐2‐6‐9‐7‐3‐8	1‐8‐5‐3‐7‐9‐6‐2 2‐7‐9‐4‐8‐6‐1‐3
9	7‐3‐5‐8‐1‐6‐2‐9‐4 6‐3‐9‐7‐2‐4‐8‐5‐1	4‐9‐5‐3‐8‐6‐1‐7‐2 2‐4‐9‐7‐1‐5‐3‐6‐8

We created 34 experimental audio‐sequences (Digit span test forward = 17 audio‐sequences; Digit span test backward = 17 audio‐sequences). For each length, there were two audio‐sequences, except for length 2. Indeed, for length 2, there were three audio‐sequences: one familiarization/example audio‐sequence followed by two otheraudio‐sequences. All audio‐sequences were divided into two playlists. The first playlist contained the nine preliminary audio‐sequences (Table 2), followed by the 17 audio‐sequences of the Digit span test forward. The second playlist included the 17 audio‐sequences of the Digit span test backward (Table 2).

##### The Bisyllabic non‐word span test

The structure and duration of the audio‐sequences in the Bisyllabic non‐word span test was the same as those in the Digit span test. The only difference was that the spoken number words were replaced by the following bisyllabic non‐words: CHEFU, CHIRE, FENU, LOMI, NORI, SELI, SIRU, VOFI, VUFO. We selected syllables that were available in an Italian database (Stella & Job, 2001). Moreover, we avoided the repetition of a syllable in the same non‐word and across non‐words, for each length. All audio‐sequences were divided into two playlists in *Windows Media Player*. The first playlist contained the nine preliminary audio‐sequences (Table 3), followed by the 17 audio‐sequences of the Bisyllabic non‐word span test forward. The second playlist included the 17 audio‐sequences of the Bisyllabic non‐word span test backward (Table 3).

**TABLE 3:** List of the non‐words included in the Bisyllabic non‐word span test (forward and backward).

Preliminary audio‐sequences							
NORI CHIRE	LOMI	VOFI	SIRU	FENU	SELI	VUFO	CHEFU

	Experimental audio‐sequences	
Length	Bisyllabic non‐word span test: Forward	Bisyllabic non‐word span test: Backward
2	SELI‐VUFO (example) SIRU‐LOMI FENU‐CHEFU	NORI‐SELI (example) SIRU‐VOFI LOMI‐CHEFU
3	CHEFU‐VOFI‐SELI NORI‐VUFO‐LOMI	CHEFU‐SIRU‐SELI VOFI‐CHEFU‐VUFO
4	CHIRE‐SIRU‐VUFO‐LOMI NORI‐LOMI‐CHEFU‐CHIRE	VUFO‐LOMI‐CHEFU‐NORI LOMI‐VUFO‐NORI‐CHIRE
5	FENU‐CHEFU‐LOMI‐SIRU‐CHIRE NORI‐SELI‐CHEFU‐CHIRE‐VOFI	NORI‐SELI‐VOFI‐VUFO‐LOMI CHIRE‐CHEFU‐LOMI‐SELI‐SIRU
6	VUFO‐VOFI‐SIRU‐FENU‐CHIRE‐SELI FENU‐VUFO‐SELI‐CHEFU‐NORI‐CHIRE	FENU‐LOMI‐CHEFU‐SIRU‐NORI‐CHIRE CHIRE‐FENU‐VUFO‐NORI‐VOFI‐SIRU
7	LOMI‐FENU‐CHEFU‐SIRU‐VUFO‐NORI‐SELI NORI‐CHIRE‐VUFO‐VOFI‐CHEFU‐SELI‐LOMI	VOFI‐VUFO‐NORI‐SIRU‐FENU‐CHIRE‐SELI SELI‐NORI‐CHIRE‐LOMI‐CHEFU‐FENU‐VUFO
8	SELI‐SIRU‐CHIRE‐LOMI‐VOFI‐CHEFU‐FENU‐NORI SIRU‐CHEFU‐NORI‐VOFI‐LOMI‐CHIRE‐VUFO‐SELI	FENU‐VOFI‐SELI‐CHIRE‐VUFO‐LOMI‐CHEFU‐SIRU VOFI‐NORI‐CHIRE‐SIRU‐SELI‐VUFO‐LOMI‐CHEFU
9	CHEFU‐VOFI‐VUFO‐CHIRE‐NORI‐FENU‐SELI‐SIRU‐LOMI VUFO‐NORI‐CHIRE‐SELI‐LOMI‐CHEFU‐FENU‐VOFI‐SIRU	LOMI‐VOFI‐NORI‐CHIRE‐CHEFU‐SELI‐FENU‐SIRU‐VUFO NORI‐CHEFU‐SIRU‐SELI‐CHIRE‐LOMI‐VUFO‐VOFI‐FENU

### Procedure

First, participants received the informed consent module and the privacy form to allow the processing of personal data. Participants were informed about the aims of the study and about their right to withdraw consent freely and at any time. In the latter case, their data would not be used for the purposes of the study. Then, participants were interviewed for the presence of the inclusion criteria. Afterwards, the participants performed the MMSE (Folstein et al., 1975; Magni et al., 1996; Measso et al., 1993). Finally, participants were administered the Digit span test and the Bisyllabic non‐word span test, in counterbalanced order. That is, half participants performed first the Digit span test and then the Bisyllabic non‐word span test. On the contrary, half participants performed first the Bisyllabic non‐word span test and then the Digit span test.

#### Digit span test procedure

##### Preliminary phase

In the preliminary phase, the participants listened to the nine audio‐sequences (tone, single spoken number word, tone). Participants had to orally repeat the spoken number word that they had just heard. For each audio‐sequence, immediately after the second tone, the examiner stopped the audio and recorded the answer of the examinee. The examiner paid particular attention to stop each audio‐sequence immediately after the second tone, because otherwise, in some cases, *Windows Media Player* would repeat the same audio‐sequence, instead of the next one. If all the preliminary spoken number words were repeated correctly, then the examiner proceeded with the experimental phase (Forward and Backward presentation).

If the participants repeated the number word incorrectly or repeated it before the second tone, the examiner repeated the instructions to the participants. The examiner could repeat the spoken number word up to three times. If the participants were still unable to repeat it correctly, they were excluded from the study. In case of one or more errors during the first presentation of the preliminary audio‐sequences, the participants were asked to listen to the entire list again (second presentation). In case of a new error, during the second presentation, the participants were excluded from the study. If, instead, all the preliminary spoken number words were repeated correctly, in the second presentation, then the examiner proceeded with the experimental phase (forward and backward modalities).

##### Experimental phase

The participants always performed first the forward modality and then the backward modality. Thus, following the procedure used by Monaco et al. (2013) and Pasotti et al. (2022), the forward and backward trials were not counterbalanced. For each presentation, the span of each participant equaled the length of the longer audio‐sequence correctly repeated by the participant, in at least one audio‐sequence for each length. For instance, the participants had a span of “four”, if they were able to repeat correctly at least one of the two sequences of length four (i.e., “6 ‐ 8 ‐ 3 ‐ 9” or “2 ‐ 1 ‐ 7 ‐ 9”), but they failed to repeat both the next sequences of length five (i.e., “3 ‐ 4 ‐ 9 ‐ 6 ‐ 8” or “7 ‐ 9 ‐ 2 ‐ 8 ‐ 1”). If participants were able to correctly perform both on the preliminary phase and on the example audio‐sequence, but then failed, they were assigned a span of “one”

###### Forward modality

The participants were administered the 17 forward audio‐sequences. First, an example of audio‐sequence was presented, consisting of two spoken number words, to allow the participants to further familiarize themselves with the task. The participants were asked to repeat the two spoken number words in the same order. If the participants repeated them correctly, they were administered the remaining 16 audio‐sequences. If the participants made an error in repeating the spoken number words or they repeated them before the second beep, the examiner repeated the stimuli and/or read again the instructions. If the participants made another mistake, they were excluded from the study. Otherwise, the participants were administered the remaining 16 audio‐sequences.

###### Backward modality

The participants were administered the 17 backward audio‐sequences. The procedure was the same as that of the forward modality, but participants were asked to repeat the spoken number words in the reverse order, from the last to the first one.

#### Bisyllabic non‐word span test procedure

The procedure was the same as for the Digit span test (forward vs. backward), but the stimuli were bisyllabic non‐words (Table 3).

#### Test–retest procedure

During the administration of the tests, 88 participants were asked to be recontacted to repeat the experimental procedure. After 7 days from the original administration (i.e. Test session), all the tests (i.e., Digit span test forward and backward; Bisyllabic non‐word span test forward and backward) were re‐administered to the participants with the aim to investigate Test–Retest reliability.

## RESULTS

We used JASP (JASP Team, 2024) to perform all statistical analyses.

### Descriptive statistics

Descriptive statistics of all spans are reported in Table 4. All raw scores are available in Supplemental Data 3 and 4 (https://osf.io/mna92/).

**TABLE 4:** Descriptive statistics of the spans.

	Digit span test forward	Digit span test backward	Bisyllabic non‐word span test forward	Bisyllabic non‐word span test backward
Valid	225	225	225	225
Missing	0	0	0	0
*M*	5.302	4.538	2.862	2.222
SE	0.077	0.082	0.054	0.039
95% CI *M* Upper	5.455	4.699	2.969	2.298
95% CI *M* Lower	5.150	4.377	2.755	2.146
SD	1.160	1.225	0.815	0.578
Minimum	2.000	1.000	1.000	1.000
Maximum	9.000	9.000	5.000	5.000

### Inferential statistics

#### Pearson's correlations: Relations between spans

**FIGURE 1:**
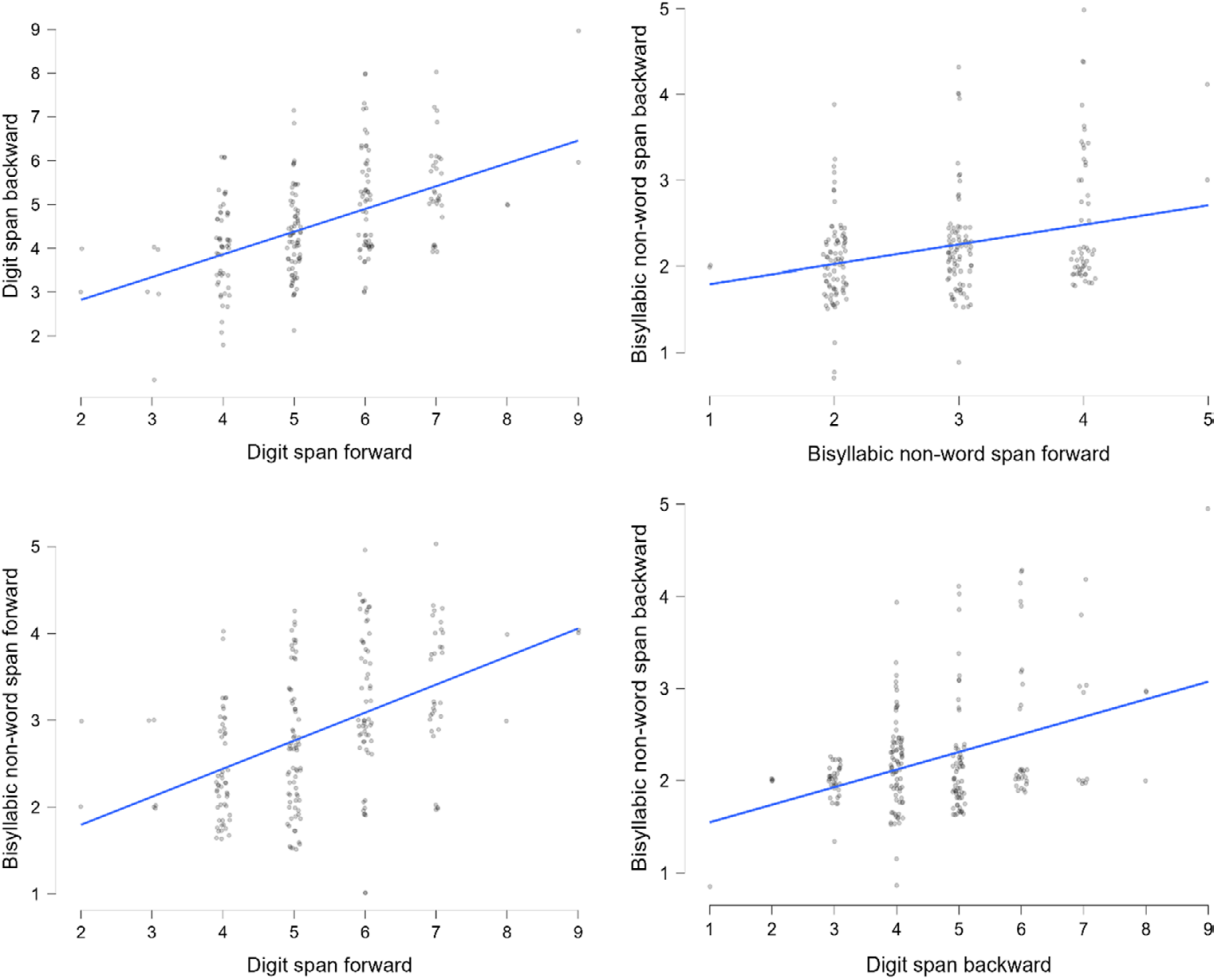
Scatterplots of the correlations between the raw scores from the four tests (i.e., Digit span forward, Digit span backward, Bisyllabic non‐word span forward, Bisyllabic non‐word span backward).

We performed Pearson's product–moment correlations between forward and backward spans (Digit span vs. Bisyllabic non‐word span; Figure 1). All correlations were positive and of medium size^1^ (see Supplemental Data 4a and 5: https://osf.io/mna92/). Pearson's *r* ranged between .321 and .492.

#### Pearson's correlations: Test–retest reliability

**FIGURE 2:**
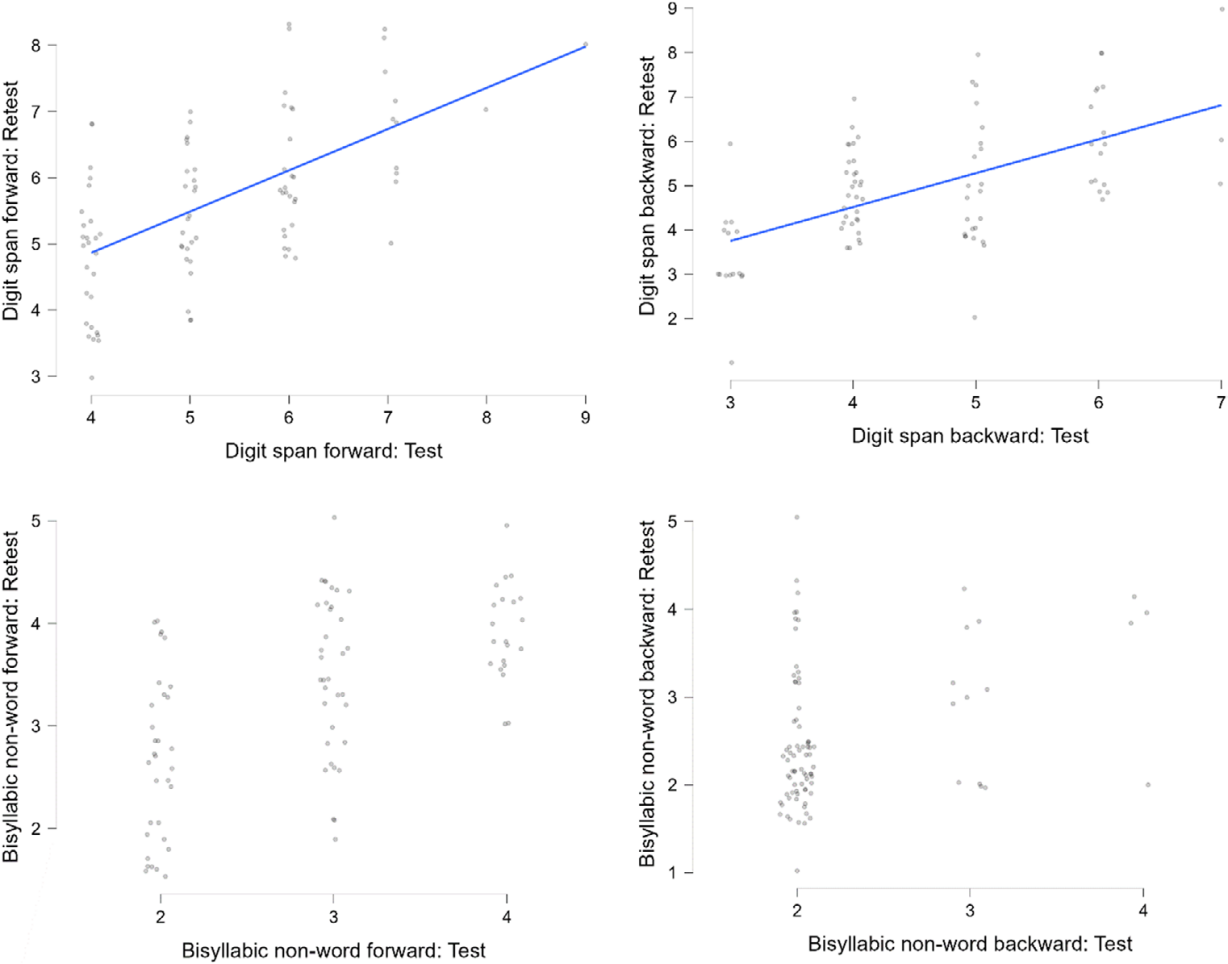
Scatterplots of the test–retest correlations between the raw scores from the four tests (i.e., Digit span forward, Digit span backward, Bisyllabic non‐word span forward, Bisyllabic non‐word span backward; Supplemental Data 4a https://osf.io/mna92/.

We investigated test–retest reliability by correlating the participants' performance on the Test session and on the Retest session (Figure 2; Supplemental Data 4a and 5 https://osf.io/mna92/). All test–retest correlations of the Digit span (forward and backward modalities) were positive and large. Pearson's *r*s ranged between .580 and .601. Finally, all correlations of the Bisyllabic non‐word span (forward and backward modalities) were positive and of medium and large size. Pearson's *r*s ranged between .355 and .598 (Figure 2; Supplemental Data 4a and 5 https://osf.io/mna92/).

#### *T*‐tests: Learning effects (test vs. retest session)

We also tested for learning effects in participants' performance between the Test and the Re‐test session. To this aim, we conducted four paired‐samples *t*‐tests corrected with Bonferroni (.05/4 comparisons; alpha = .0125; Table 5; Supplemental Data 4a and 5 https://osf.io/mna92/). In all the comparisons, the participants' performance was significantly better on the Re‐test than on the Test session.

**TABLE 5:** Comparison between test and re‐test sessions.

Measure 1		Measure 2	*t*	df	*p*	*M* difference	SE difference	95% CI for *M* difference		Cohen's *d*	SE Cohen's *d*	95% CI for Cohen's *d*	
								Lower	Upper			Lower	Upper
Test: Digit span forward	‐	Retest: Digit span forward	−3.547	87	<.001	−0.386	0.109	−0.603	−0.170	−0.378	0.099	−0.593	−0.161
Test: Digit span backward	‐	Retest: Digit span backward	−3.050	87	.003	−0.386	0.127	−0.638	−0.135	−0.325	0.100	−0.539	−0.110
Test: Bisyllabic non‐word span forward	‐	Retest: Bisyllabic non‐word span forward	−5.582	87	<.001	−0.420	0.075	−0.570	−0.271	−0.595	0.104	−0.820	−0.367
Test: Bisyllabic non‐word span backward	‐	Retest: Bisyllabic non‐word span backward	−3.264	87	<.001	−0.273	0.084	−0.439	−0.107	−0.348	0.125	−0.562	−0.132

*Note*: Student's *t*‐test. Alpha corrected with Bonferroni = .0125.

#### *T*‐tests: Comparison between spans

We performed paired‐samples *t*‐tests to compare mean forward and backward spans (digits vs. bisyllabic non‐words), corrected with Bonferroni (.05/4 comparisons; alpha = .0125). All differences were significant (Table 6). Indeed, mean Digit spans were higher than mean Bisyllabic non‐word spans (Table 6). Mean forward spans were higher than mean backward spans (Table 6). Effects sizes ranged from medium (Digit span forward vs. Digit span backward) to large (Digit span forward vs. Bisyllabic non‐word span forward). Bar plots are available in Supplemental Data 5 (https://osf.io/mna92/). Note, however, that on the Digit span test, approximately 15% of the participants performed better on the backward modality than on the forward one. Furthermore, 7% of the participants showed the same pattern on the Bisyllabic non‐word span test.

**TABLE 6:** Paired‐samples *t*‐tests between spans.

Measure 1		Measure 2	*t*	df	*p*	*M* difference	SE difference	95% CI for *M* difference		Cohen's *d*	SE Cohen's *d*	95% CI for Cohen's *d*	
								Lower	Upper			Lower	Upper
Digit span forward	‐	Digit span backward	9.527	224	<.0001	0.764	0.080	0.606	0.923	0.635	0.074	0.491	0.778
Bisyllabic non‐word span forward	‐	Bisyllabic non‐word span backward	11.509	224	<.0001	0.640	0.056	0.530	0.750	0.767	0.088	0.618	0.915
Digit span forward		‐Bisyllabic non‐word span forward	34.274	224	<.0001	2.440	0.071	2.300	2.580	2.285	0.132	2.036	2.531
Digit span backward	‐	Bisyllabic non‐word span backward	30.923	224	<.0001	2.316	0.075	2.168	2.463	2.062	0.129	1.830	2.292

*Note*: Student's *t‐*test. Alpha corrected with Bonferroni = .0125.

Finally, to control for administration order effects, we conducted four *t‐*tests for independent measures, with order as the independent variable (Order 1. First test administered: Digit span; Second test administered: Bisyllabic non‐word span; Order 2. First test administered: Bisyllabic non‐word span; Second test administered: Digit span). The dependent variable was span (Digit span forward, Digit span backward, Bisyllabic non‐word span forward, Bisyllabic non‐word span backward). All the four differences were not significant (Table 7. Supplemental Data 4b https://osf.io/mna92/).

**TABLE 7:** Independent‐samples *t*‐tests between spans for order.

	Statistic	df	*p*	*M* Difference	SE Difference	95% CI for *M* difference		Cohen's *d*	SE Cohen's *d*	95% CI for Cohen's *d*	
						Lower	Upper			Lower	Upper
Digit span forward	1.679	223	.095	0.259	0.154	−0.045	0.562	0.224	0.134	−0.039	0.486
Digit span backward	−0.686	223	.493	−0.112	0.163	−0.434	0.210	−0.091	0.133	−0.353	0.170
Bisyllabic non‐word span forward	−0.044	223	.965	−0.005	0.112	−0.226	0.216	−0.006	0.133	−0.267	0.255
Bisyllabic non‐word span backward	1.033	223	.303	0.083	0.081	−0.076	0.242	0.138	0.134	−0.124	0.399

#### Multiple linear regressions

To predict the raw scores of the participants, for each span (criterion), we performed multiple linear regressions with the following predictors: Age in years (including the transformations: LOG10, natural logarithm: ln, and ln 100‐Age), Education in years (including the transformations: squared root, inverse) and Biological sex (males vs. females). For each span, the best linear regression model was the one with the highest number of significant predictors and the highest amount of explained variance for multiple predictors (i.e., Adjusted *R* ^2^; but see Arcara, 2024, for a new approach). The full range of linear regression models is reported in Supplemental Data 5 (https://osf.io/mna92/).

##### Digit span test (forward)

The overall regression was significant, Adjusted *R* ^2^ = .186, *F*(3, 221) = 18.093, *p* < .0001. We corrected *p*‐values with Bonferroni (.05/3 predictors; alpha = .0166). Age (LOG10; *b* = −1.815, *p* < .0001) and Education (*b* = 0.047, *p* = .01) were significant predictors of the Digit span forward. By contrast, Biological sex was not a significant predictor. To transform raw scores into adjusted scores we used the following equation:

$$\text{Adjusted score}=\mathrm{Raw}\;\text{score}+1.815\;X\;\left(\mathrm{LOG}10\left(\mathrm{Age}\right)-1.677\right)-0.047\;X\;\left(\text{Education}-12.569\right)$$

##### Digit span test (backward)

The overall regression was significant, Adjusted *R* ^2^ = .291, *F*(3, 221) = 31.635, *p* < .0001. We corrected *p*‐values with Bonferroni (.05/3 predictors; alpha = .0166). Age (LOG10; *b* = −1.770, *p* < .0001) and Education (squared root; *b* = 0.539, *p* < .0001) were significant predictors of the Digit span backward. By contrast, Biological sex was not a significant predictor. To transform raw scores into adjusted scores we used the following equation:

$$\text{Adjusted score}=\mathrm{Raw}\;\text{score}+1.77\;X\;\left(\mathrm{LOG}10\left(\mathrm{Age}\right)-1.677\right)-0.539\;X\;\left(\text{Squared root}\left(\text{Education}\right)-3.462\right)$$

##### Bisyllabic non‐word span test (forward)

The overall regression was significant, Adjusted *R* ^2^ = .217, *F*(3, 221) = 21.732, *p* < .0001. We corrected *p*‐values with Bonferroni (.05/3 predictors; alpha = .0166). Only Age (*b* = − 0.017, *p* < .0001) was a significant predictor of the Bisyllabic non‐word span forward. By contrast, Education and Biological sex were not significant predictors. To transform raw scores into adjusted scores we used the following equation:

$$\text{Adjusted score}=\mathrm{Raw}\;\text{score}+0.017\;X\;\left(\mathrm{Age}-51.56\right)$$

##### Bisyllabic non‐word span test (backward)

The overall regression was significant, Adjusted *R* ^2^ = .073, *F*(3, 221) = 6.882, *p* < .0001. We corrected *p*‐values with Bonferroni (.05/3 predictors; alpha = .0166). Only Age (LOG10; *b* = −0.738, *p* < .01) was a significant predictor of the Bisyllabic non‐word span backward. By contrast, Education and Biological sex were not significant predictors. To transform raw scores into adjusted scores we used the following equation:

$$\text{Adjusted score}=\mathrm{Raw}\;\text{score}+0.738\;X\;\left(\mathrm{LOG}10\left(\mathrm{Age}\right)-1677\right)$$

#### Adjusted and equivalent scores

From raw scores, we calculated adjusted scores, for all spans, through the spreadsheets available in Supplemental Data 6 (https://osf.io/mna92/). Adjusted scores were then ordered from the smallest to the highest. Then we calculated equivalent scores, by means of this software: https://egdp.shinyapps.io/tolLimits/ (Aiello & Depaoli, 2022). Equivalent scores corresponded to the following observations (see also Supplemental Data 6 https://osf.io/mna92/):

- The observation corresponding to the outer limit (equivalent score 0) was the 6th with safety level 96.9%;
- The observation corresponding to the inner limit was the 18th with a safety level 96.8%;
- The last observation corresponding to an equivalent score equal to 1 was the 22nd;
- The last observation corresponding to an equivalent score equal to 2 was the 58th;
- The last observation corresponding to an equivalent score equal to 3 was the 112th.

For each span, the range of the adjusted scores, within each equivalent score (0–4), is reported in Supplemental Data 6 (https://osf.io/mna92/). Starting from the raw scores, clinicians can use this spreadsheet to calculate the adjusted scores. Finally, the adjusted scores can be classified into the 0–4 scale of the equivalent scores (0 = abnormal performance; 1 = borderline performance; 2–4: normal performance (Aiello & Depaoli, 2022; Capitani & Laiacona, 2017). When the same adjusted score was observed at the threshold between two equivalent scores, the last observation within the lower equivalent score was shifted to the next smaller adjusted score for equivalent score 0, but to the next larger adjusted score for equivalent scores 1–4 (Aiello & Depaoli, https://osf.io/mna92/ 2022). A video explaining the use of the spreadsheets to calculate adjusted and equivalent scores is available (Supplemental Video https://osf.io/mna92/).

## DISCUSSION

We aimed to standardize two tests measuring the capacity of the phonological loop (forward modality) and the contribution of the central executive (backward modality). We standardized a new version of the Digit span test and the first Italian version of the Bisyllabic non‐word span test. The introduction of computerized tools addresses key limitations of previous standardizations and provides tests with improved reliability and validity. As we mentioned, we introduced the use of non‐words, as a specific measure of the phonological loop. On both tests (i.e., Digit span and Bisyllabic non‐word span), we added a single‐stimulus preliminary test for sensory‐perceptual/articulatory‐motor screening. This approach ensures that other factors, such as sensory‐perceptual/articulatory‐motor impairments, do not interfere with the test performance.

For all tests, construct validity was supported by the significant correlations among the tests (often large). Moreover, a significant advancement of the present study is the first evaluation of the reliability of both the Digit span and the Bisyllabic non‐word span tests, through a test–retest procedure. Indeed, in the previous standardizations (Monaco et al., 2013; Orsini et al., 1987; Pasotti et al., 2022) test–retest reliability was not tested.

Phonological short‐term memory, although is a function that can be continuously trained and improved, typically does not exhibit substantial variability over short periods. In our study, Test–Retest reliability was supported by the presence of significant correlations between scores in the Test session and in the Retest session. Our results confirm the actual reliability of both tests, with a large correlation between the initial (Test session) and subsequent (Retest session) administrations of the protocol. Note, however, that we also observed learning effects between the two sessions (Test vs. Retest). Indeed, participants' performance was significantly higher in the Retest than in the Test session, for all the four tests (i.e., Digit span forward, Digit span backward, Bisyllabic non‐word span forward, Bisyllabic non‐word span backward). These learning effects should be carefully considered by clinicians, who plan to repeatedly administer the tests to the same patient. Finally, we hypothesize that without these learning effects, rest‐retest reliability might have been even higher.

It is worth noting that on the Digit span test, approximately 15% of the participants performed better on the backward modality than on the forward one. Moreover, 7% of the participants showed the same pattern on the Bisyllabic non‐word span test. This is a rare, but not unusual finding. Indeed, there is some evidence in the literature, both in children and adults (Hoadley et al., 2013; Pham & Archibald, 2023). This effect is probably due to random attentional fluctuations (e.g., paying more attention during the backward than during the forward modality). Nevertheless, Hoadley et al. (2013) have suggested that a higher backward than forward span might be shown by individuals with better cognitive performance.

For the Digit span test, our results confirmed those of the previous standardizations (Monaco et al., 2013; Orsini et al., 1987) reporting that Age and Education, but not Biological sex (but see, Pasotti et al., 2022, only for the Digit span backward) were significant predictors of the examinees' performance. Thus, raw scores should be corrected to obtain adjusted scores for Age and Education, to better control for the impact of the predictors upon the examinees' performance. In this way, the contribution of the demographic/biological variables can be taken into account together with the contribution of a brain lesion affecting the phonological loop.

For the Bisyllabic non‐word span test, the only significant predictor was Age. Indeed, Biological sex and Education did not significantly predict the examinees' performance. The absence of a correlation between Education and the Bisyllabic non‐word span test further suggests that the contents of long‐term memory, acquired through experience and education (in other words, the use of strategies to improve performance), do not influence the Bisyllabic non‐word span. Hence, the new Bisyllabic non‐word span test –through the use of novel, non‐lexical stimuli that participants are unlikely to have encountered before– overcomes potential influences by individuals' prior knowledge, education level, and stimuli received from their environment. This allows the identification of specific cognitive deficits that might be masked on other tests by an individual's preexisting knowledge and educational experiences.

We suggest that the Bisyllabic non‐word span test could be a less‐strategy prone measure of the phonological loop. Indeed, the mean Digit span (i.e., possible influence of long‐term memory) was approximately twice as large as the Bisyllabic non‐word span (no influence from long‐term memory). This finding reinforces the hypothesis that the Bisyllabic non‐word span test is a more sensitive measure of the phonological loop, by minimizing external variables that could affect performance. Finally, all forward spans were significantly larger than the backward spans suggesting, in the latter, a resource‐consuming involvement of the central executive.

The administration of the Digit span and Bisyllabic non‐word span tests through a computerized presentation ensured an identical administration for all examinees and minimized all possible influences of the examiner. Conversely, in the classic method of test administration (i.e., the examiner reads aloud the stimuli), there are several, uncontrolled (and uncontrollable) sources of variability that are linked to differences among examiners in the voice, in the prosody, in the rhythm, and in the duration of presentation of the stimuli, including the pace at which stimuli are presented. With our computerized presentation, all those limits are completely ruled out. This standardization of administration modalities provides a more consistent evaluation of the phonological loop function and working memory. We suggest that computerized neuropsychological testing should become the gold standard for controlling the precision of stimuli presentation (Bauer et al., 2012; Harris et al., 2024).

Performance on tests measuring the phonological loop can be compromised not only because of deficits of the phonological loop, but also because of sensory (e.g., deafness) or articulatory deficits (e.g., speech apraxia, dysarthria). The use of our preliminary, screening test allowed us to exclude any influence of sensory‐perceptual and articulatory‐motor processes on the performance of the examinees. This initial screening step is crucial to ensure that test results were free from extraneous variables.

We suggest that, together with the Digit span test, the Bisyllabic non‐word span test is a useful measure of the phonological loop. Because the Bisyllabic non‐word span test is a less strategy‐prone measure than the Digit span test (with limited possibilities to adopt memory strategies), we highly recommend its use to assess neuropsychological deficits of the phonological loop. These deficits can be detrimental for patients who must learn new phonological information (e.g., acronyms, neologisms, foreign languages; Baddeley et al., 1998; Papagno et al., 1991) or must process syntactically complex sentences (Papagno et al., 2007). Indeed, if these patients are tested with the Digit span test only, they might compensate for their difficulties through the use of strategies that tap long‐term memory knowledge and/or mental imagery; this is not (or is unlikely) the case with the Bisyllabic non‐word span test.

The use of numerical digits involves information that has generally been assimilated since childhood and engages multiple memory structures, such as long‐term memory. In contrast, the use of stimuli like bisyllabic non‐words allows for the exclusive investigation of the participants' phonological short‐term memory capacity, as they are exposed to those stimuli only at the time of administration, having never learned them before.

One limit of our study is that we did not precisely and specifically control the construction of our non‐words for variables, such as the frequency of some syllables or the transition probability across syllables. This limit should be addressed in future studies. Another limitation is that we did not use a standard test to assess whether participants had any auditory impairments, such as pure‐tone audiometry or a similar audiological measurement. Nevertheless, audiological measurement is typically unavailable in standard clinical neuropsychology services. Therefore, we opted for a fixed sound intensity that was sufficiently high to be perceived by a listener without significant hearing impairment. Additionally, we chose to use headphones to mitigate the potentially detrimental effects of environmental noise and to help participants focus solely on the presented sound stimuli. Finally, we conducted a preliminary test to ensure that all acoustic stimuli were correctly perceived by the participants.

A final limit of our study is that we were able to recruit very few participants who were older than 85 years (i.e., range 85–89 = 7 participants; ≥90 = 3 participants). Nevertheless, we believe that regression models, such as the ones we used, are powerful enough to predict values even if people older than 85 were not widely represented (see also Pasotti et al., 2022).

To compare the results of the various standardizations of the Digit span test, we used the performance of a hypothetical 55‐year‐old male, with 8 years of education, who had a raw score of five (span). The raw score of this participant was then corrected according to the four Italian standardizations that included the Digit span forward modality (Monaco et al., 2013; Orsini et al., 1987; Pasotti et al., 2022, and the present standardization).

Important differences were observed among the standardizations. For instance, the participant could obtain an equivalent score of 1 (Monaco et al., 2013), 2 (Pasotti et al., 2022) or 4 (Orsini et al., 1987 and the present standardization). One can reason that this might be due to differences in the stimuli used in the four standardizations, but this is not plausible. Indeed, Monaco et al. (2013), Orsini et al. (1987), and Pasotti et al. (2022) used the stimuli of the WAIS‐R (Wechsler Adult Intelligence Scale‐Revised; Orsini & Laicardi, 1998). In contrast, in the present standardization, we created our own stimuli. Thus, different equivalent scores can be obtained when the same stimuli are used (Orsini et al., 1987 vs. Monaco et al., 2013; Pasotti et al., 2022), but the same equivalent score can be obtained when different stimuli are used (e.g., Orsini et al., 1987 vs. the present standardization).

One might argue that the differences, among the standardizations, can be observed because the standardizations of Orsini et al. (1987) and the present one included less participants than the other ones (Monaco et al., 2013; Pasotti et al., 2022); this does not seem to be the case. Indeed, Orsini et al. (1987) and we recruited a total of 1580 participants, whereas in the other two standardizations (Monaco et al., 2013; Pasotti et al., 2022) a total of 530 participants were recruited. Furthermore, one might argue that there can be socio‐cultural and technological effects in more recent standardizations (Pasotti et al., 2022 and the present one) than in the older ones (Monaco et al., 2013; Orsini et al., 1987); even this, however, does not seem to be the case. Indeed, there are differences between the two most recent standardizations (Pasotti et al., 2022 vs. the present study), but not between our standardization and the oldest one (Orsini et al., 1987).

Monaco et al. (2013) have hypothesized that the differences between their standardization and that of Orsini et al. (1987) might be due to the higher education level of their sample (mean education: 11.63 years) than that of Orsini et al. (1987; mean education: 9.44 years). Nonetheless, this difference was even higher when the sample of Orsini et al. (1987) was compared with that of the present standardization (9.44 vs. 12.56 years), but the results were similar.

Very surprisingly, when we compared the performance of the hypothetical participant on the three standardizations that included the backward version of the Digit span test (Monaco et al., 2013; Pasotti et al., 2022, and the present standardization), the participant always obtained the same equivalent score (i.e., 4). Thus, it seems that the differences among the standardizations regard exclusively the Digit span forward. We suggest that future ad hoc studies are necessary to explain the differences among the various standardizations in terms of demographic, methodological, and statistical issues.

Future studies can further validate the Bisyllabic non‐word span test, on patients, to assess its diagnostic utility in clinical populations, such as individuals with neurological disorders, focal brain lesions and cognitive impairments, and to determine its sensitivity for early detection of specific memory and executive disorders. Advanced neuroimaging techniques could also provide more detailed insights into the brain regions and neural pathways involved in the phonological loop and central executive functions. Longitudinal studies tracking changes in phonological short‐term memory over extended periods can provide insights into the tests' ability to detect subtle cognitive changes or improvements from interventions.

In conclusion, the proposed protocol can be a tool with good validity and adequate reliability for both researchers and clinicians. The Bisyllabic non‐word span test could be an innovative and specific measure for identifying phonological short‐term memory deficits, monitoring memory performance over time, and designing personalized neuropsychological rehabilitation programs based on the scores obtained by individual patients.

## AUTHOR CONTRIBUTIONS

**Konstantinos Priftis:** conceptualization; investigation; methodology; writing—original draft; Writing—review & editing; formal analysis; supervision; project administration. **Daphne Gasparre:** investigation; writing—review & editing; formal analysis; data curation; validation. **Denyse Carazzolo:** investigation; writing—review & editing; data curation; validation. **Valeria Vaccaro:** data curation; writing—review & editing; investigation; validation. **Roberta Toffano:** writing—review & editing; data curation; validation. **Marco Pitteri:** conceptualization; writing—review & editing; supervision. **Massimo Grassi:** conceptualization; investigation; data curation; writing—review & editing.

## CONFLICT OF INTEREST STATEMENT

No funds, grants, or other support was received for the present study. The authors have no financial or non‐financial interests to disclose.

## Data Availability

The data that support the findings of this study are openly available in Open Science Framework at https://osf.io/mna92/.

## Footnotes

^1^
We used Cohen's criteria (Cohen, 1988) to evaluate the size of *t*‐tests and correlation coefficients.
